# Dr. George R. Bartran

**Published:** 1913-11

**Authors:** 


Dr. George R. Bartran, Chicago Medical College, 1872, who had practiced for thirty-four years at Algoma, Wis., died at his old home in Smithboror, N. Y., August 27, aged 72.

